# Time Synchronization of Multimodal Physiological Signals through Alignment of Common Signal Types and Its Technical Considerations in Digital Health

**DOI:** 10.3390/jimaging8050120

**Published:** 2022-04-21

**Authors:** Ran Xiao, Cheng Ding, Xiao Hu

**Affiliations:** 1 School of Nursing, Duke University, Durham, NC 27708, USA; 2 Department of Biomedical Engineering, Georgia Institute of Technology, Emory University, Atlanta, GA 30332, USA; chengding@gatech.edu; 3 School of Nursing, Emory University, Atlanta, GA 30322, USA; xiao.hu@emory.edu; 4 Department of Biomedical Informatics, School of Medicine, Emory University, Atlanta, GA 30322, USA; 5 Department of Computer Science, College of Arts and Sciences, Emory University, Atlanta, GA 30322, USA

**Keywords:** time alignment, digital health, multimodal signals, cross-correlation

## Abstract

Background: Despite advancements in digital health, it remains challenging to obtain precise time synchronization of multimodal physiological signals collected through different devices. Existing algorithms mainly rely on specific physiological features that restrict the use cases to certain signal types. The present study aims to complement previous algorithms and solve a niche time alignment problem when a common signal type is available across different devices. Methods: We proposed a simple time alignment approach based on the direct cross-correlation of temporal amplitudes, making it agnostic and thus generalizable to different signal types. The approach was tested on a public electrocardiographic (ECG) dataset to simulate the synchronization of signals collected from an ECG watch and an ECG patch. The algorithm was evaluated considering key practical factors, including sample durations, signal quality index (SQI), resilience to noise, and varying sampling rates. Results: The proposed approach requires a short sample duration (30 s) to operate, and demonstrates stable performance across varying sampling rates and resilience to common noise. The lowest synchronization delay achieved by the algorithm is 0.13 s with the integration of SQI thresholding. Conclusions: Our findings help improve the time alignment of multimodal signals in digital health and advance healthcare toward precise remote monitoring and disease prevention.

## 1. Introduction

The last decade has witnessed a rapid expansion in the field of digital health, driven by the burgeoning portable and wearable sensor technologies, medical devices based on Internet of Things (IoT), and advancement in big data analytics through AI and machine learning [1,2,3,4,5]. The field is further accelerated by the development of big data service platforms that provide the infrastructure for large-scale data storage and analysis [6]. Digital health, or mobile health (mHealth), is gradually transforming the clinical management of chronic diseases, such as diabetes mellitus and heart diseases, which require close and continuous monitoring between clinical visits [7,8], as well as fostering telehealth that is accelerated in the era of COVID-19 [9,10]. The recent advances and wide adoption of wearable and wireless sensor technologies, such as fitness trackers, wireless patches, and smartwatches, bring about large volumes of patient-generated health data (PGHD) that are user-initiated and include the continuous multimodal monitoring of physiological conditions. By leveraging big data analytical tools, particularly machine learning and deep learning, digital health helps shift healthcare toward prevention and the early detection of diseases [5,11,12]. 

Despite the advantages and convenience of monitoring various physiological conditions offered by digital health innovations, one challenge that arises is how to synchronize the timestamps of multimodal physiological signals across different devices. The precise temporal synchronization between physiological signals has a significant impact on downstream applications. First, it is the mainstay for validating emerging technologies with established devices as the gold standard. Novel features and data modalities are constantly being introduced to expand the digital health arsenal. Validation studies are performed to confirm their accuracy by comparing them to data collected simultaneously from standard medical-grade devices [13,14,15,16,17]. Being able to correctly match the timestamps of different signals is the foundation for the validation. Second, an increasing number of studies leverage complementary information in multimodal physiological signals to make new discoveries and improve system performance in various tasks [18,19,20,21]. Precise time alignment is again the foundation to build these synchronous multimodal datasets.

Achieving precise multimodal signal synchronization is challenging in that the signal morphology might be different from one another, making it difficult to define the similarity metric for the time-matching algorithms [22]. In addition, signals from different devices lack the ground truth to validate the algorithm performance. Several studies have been carried out aiming to provide a time alignment solution for multimodal physiological signals. Jiang et al. employed a dynamic time warping (DTW) approach, termed eventDTW, which uses information from defined events (i.e., upslope and downslope in the signal) as the basis to find the optimal time alignment between two signals [23]. It is known that the DTW algorithm is susceptible to the singularity problem when two time series are measured at different sampling rates [24,25]. The authors alleviate this issue by leveraging local information in the signal from the defined events and demonstrate improved performance over two other state-of-the-art DTW algorithms, especially when aligning two signals measured with different frequencies [25,26]. Liu et al. proposed another novel approach, termed Phyio2Video, which involves spectral feature extraction from different physiological signals, and converts them into feature videos so that the signal alignment task is transformed into a video frame alignment task [22]. To tackle the video frame alignment task, the authors used the deep convolutional neural network (CNN) to obtain nonlinear encoding of the feature videos, which determine the final alignment of two signals by implementing a canonical correlation loss.

The above studies achieved inspiring results in aligning two signals of different modalities. However, their advantage in aligning signals of the same type is not clear. In addition, most existing algorithms have not been tested with consideration of various factors associated with digital health technologies that might be detrimental to the algorithm performance. The present study aims to complement previous studies by solving a specific but prevalent signal time-alignment problem in digital health when a common signal type is available across different devices. We propose a time alignment approach that is based on simple temporal cross-correlation of two time series to obtain the optimal time alignment. To demonstrate the proposed approach, two of the 12-lead data from a public arrhythmia database are adopted to simulate synchronous data collection with a smartwatch and an ECG patch. This setup not only offers ground truth to gauge the performance of the proposed time-alignment algorithm but also provides a more realistic simulation when pathological conditions are present in the signal. A series of experiments are carried out to comprehensively evaluate the algorithm performance and study the impact of key factors involving the time-alignment process, including minimal signal duration, integration of signal quality index, the impact of signal noise, and different sampling rates. Algorithm performance under these controlled experiments is compared and reported, based on which we provide practical recommendations with respect to the above factors in time-alignment tasks.

## 2. Materials and Methods

### 2.1. Study Data

The data used in the study are from a publicly available dataset on PhysioNet, the St. Petersburg Institute of Cardiological Technics (INCART) 12-lead Arrhythmia Database [27]. The database contains 75 30 min continuous 12-lead ECG recordings, which are segmented from 32 Holter records. The dataset was recorded with a sampling rate of 257 Hz. The ECG data were collected from patients (46.9% female; mean age at 58 years with an age range: 18–80 years) under suspicion of coronary artery diseases. Clinical annotations of cardiac arrhythmias, such as atrial fibrillation, bradycardia, etc., and final patient diagnosis were provided for each recording in the dataset (refer to PhysioNet for a complete list [27]). The database was selected because it contains ample continuous recordings to extensively test the proposed algorithm, and it covers a broad spectrum of ECG abnormities that can provide a critical evaluation of the algorithm when irregular heartbeats exist in the signal. This study received an exemption from the Institutional Review Board (IRB) at Duke University, as it uses de-identified and publicly available data from PhysioNet. 

### 2.2. Sample Selection

In the present study, we selected 2 of the 12-lead data available in the dataset, including a limb Lead I and a precordial Lead V2. The choice made sense in simulating the scenario of synchronous data collection with the smartwatch and the ECG patch. The smartwatch worn on the wrist measures ECG signals assimilating to the clinical Lead I. For the ECG patch, when installed in recommended vertical placement on the sternum, it mostly represents the clinical V2 lead. For each recording, 100 m-second segments of data were randomly selected as Lead I samples. The choice of duration (m) for Lead I samples is a factor to investigate in Experiment #1, which is described in detail below. For each sample from Lead I, a segment in Lead V2 with matched timestamps and duration is selected, which is combined with 30 s data both before and after the segment to form the Lead V2 sample. Therefore, each test sample consists of a Lead I sample and its matched Lead V2 sample which is 60 s longer in duration. Since data from both leads are simultaneously recorded in the dataset, the matched time stamp serves as the ground truth. A deviation in time (as determined by the proposed algorithm) from the ground truth is termed the sync delay, which serves as the metric to evaluate the algorithm performance. In total, there are 7500 test samples from all 75 recordings in the INCART database included in the study. 

### 2.3. Time Alignment Algorithm

The proposed algorithm for synchronizing timestamps of cross-device multimodal data takes advantage of physiological signals (i.e., ECG in the present study) commonly available across devices as the gateway. The algorithm consists of three main components, including signal preprocessing, sync delay estimation, and the integration of the signal quality index (SQI). Both environmental (e.g., DC and powerline noise) and technical factors (e.g., change of impedance, and loose lead contact) can negatively impact the quality of acquired signals, so we deployed a series of signal preprocessing steps as the first component of the algorithm to help mitigate the signal quality issue through improving the signal-to-noise ratio (SNR). The signal was downsampled to a more commonly used sampling frequency at 250 Hz to facilitate the evaluation of different sampling rates’ impact on algorithm performance (see details in Experiment #4 below) and to reduce the computational cost. A second-order Butterworth bandpass filter (2–10 Hz) was applied to the resampled signals to reduce baseline wandering and high-frequency noise. Backward–forward filtering was implemented to avoid any phase delay in the filtered signal, which is a critical consideration in designing time synchronization algorithms [28]. Lastly, min-max normalization was applied to signals from each ECG lead to bring data of different leads to an equal footing.

The second component of the time alignment algorithm is to estimate the sync delay between signals from the two different ECG leads. This is achieved by estimating the cross-correlation between Lead I signal $S_{I}$ and Lead V2 signal $S_{V2}$. As shown in the pseudocodes in Algorithm 1, time lags are introduced in $S_{V2}$ to generate the shifted signal ${\tilde{S}}_{V2}$ in both positive and negative temporal directions with a step of one sample in the signal. Correlation coefficients are calculated between $S_{I}$ and each shifted Lead V2 signal ${\tilde{S}}_{V2}$. Finally, the sync delay is the time lag that corresponds to the largest correlation coefficient between two signals. The correlation $r_{i}$ at *i*-th time lag $t_{i}$ between Lead I signal $S_{I}$ and time-shifted Lead V2 signal ${\tilde{S}}_{V2}$ is calculated by

(1) $$r_{i}=E\left(S_{I}\cdot {\tilde{S}}_{V2_{i}}^{*}\right)$$

where E is the expected value operator and ${\tilde{S}}_{V2_{i}}^{*}$ is the complex conjugation of the time-shifted Lead V2 signal at the *i*-th time lag. The cross-correlation operation is carried out using the “xcorr” function in MATLAB (ver. 2021a). An index table for notations in the time alignment algorithm and key terminologies in the manuscript is provided in Supplementary Table S1.
**Algorithm 1.** Algorithm for synchronization delay estimation
 **input**: Lead I signal $S_{I}$, Lead V2 signal $S_{V2}$
 **output**: synchronization delay τ1 **for** every time lag $t_{i}$ that shifts $S_{V2}$ to either positive or negative direction **do**2    compute correlation coefficient $r_{i}$ between $S_{I}$ and shifted signal ${\tilde{S}}_{V2}$3 end4 compute maximal $r_{i}$ and find corresponding time lag $t_{max}$5 **return**
$\tau =t_{max}$

The last component of the algorithm is to establish a signal quality index (SQI) for each ECG sample. This component provides an option to remove samples compromised by poor quality signals and investigate whether that can improve the performance of the proposed algorithm (see Experiment #2 below). The present study adopts a dynamic SQI (dSQI) that is based on template matching of time–frequency features in ECG signals and offers reliable quality assessment for ECGs with either normal sinus rhythm or common arrhythmias [29]. The quality assessment algorithm first derives the time–frequency representation of each ECG beat using a smoothed pseudo Wigner–Ville transform (SPWVD), which provides improved time–frequency localization, compared to the more commonly used short-time Fourier transform (STFT) [30]. For every three beats in the signal, the algorithm generates a template by averaging the time–frequency representation of these beats. A cross-correlation is computed between the template and the fourth upcoming beat (i.e., the target beat) to generate the SQI for the target beat. In this way, the beat-to-beat SQI can be derived for the entire signal. The present study then uses the total quality score that is defined as the percentage of good-quality beats across all beats in one sample as the sample SQI. All analyses in the study were performed using MATLAB (MathWorks Inc., Natick, MA, USA).

### 2.4. Experiment Setup

A series of experiments are conducted to evaluate the performance of the proposed algorithm and to investigate the impact of multiple key factors on the time alignment performance. These factors include the sample duration (m) of Lead I samples, the SQI-based threshold for noisy sample removal, the noise type and level in ECG, and the signal sampling rate. 

#### 2.4.1. Experiment #1 Sample Duration Test

The duration of Lead I sample directly affects the efficiency of the time–synchronization algorithm, i.e., the shorter the sample duration needed, the faster the algorithm can compute, the less computing resource is required, and the greater the convenience for users to collect the data. However, its impact on the algorithm performance is unknown. There might exist a balancing point between efficiency and performance for the choice of sample duration. Therefore, the experiment is designed to use different sample durations for Lead I samples and probe the corresponding performance of the algorithm. The sample durations being tested range from 10 s to 50 s with an increment of 10 s. All choices are confined to under one minute to make the algorithm more practically manageable to end-users. For each choice of sample durations, a matched set of Lead V2 samples is created by extracting an additional 60 s of signals in Lead V2, as described in Section 2.2. The time alignment algorithm is applied to all five sample groups to derive corresponding sync delays. The algorithm performance is compared across different groups, and one duration is determined with consideration of both algorithm efficiency and performance for the subsequent experiments. The analysis of variance (ANOVA) test is adopted to test whether there is a significant difference in the algorithm performance across the five groups. A post hoc Tukey’s HSD (honestly significant difference) test is then performed for pairwise comparisons among sample groups. The significant level α is set at 0.05 for all statistical tests in the study. 

#### 2.4.2. Experiment #2 Integration of SQI

Signal quality is the backbone of a reliable time-alignment algorithm. Aside from adopting a list of preprocessing steps to improve the signal-to-noise ratio of ECG signals, an overall quality assessment of the entire sample signal can serve as an additional gatekeeper to filter out samples with relatively low signal quality where the algorithm may perform poorly. In this experiment, SQI thresholds from 0 (retaining all samples regardless of their signal quality) to 1 (retaining only samples with perfect signal quality) with an increment of 0.1 are selected to remove samples failing the quality thresholding criteria and to establish the corresponding algorithm performance. A one-way ANOVA test is performed to test whether the SQI threshold is significantly associated with the algorithm performance. To control for the effect of SQI, the set of samples with SQI over the threshold of one is selected for subsequent experiments. 

#### 2.4.3. Experiment #3 Noise Stress Test

The third experiment is dedicated to evaluating the algorithm performance when common ECG noise types are present in the signal. Three noise types prevalent in ECG signals are investigated, including baseline wandering (BW), electrode motion artifact (EM), and muscle artifact (MA). Different noise types are artificially added to the ECG samples in the present study using the WFDB software and the MIT-BIH noise stress test database, both freely accessible on PhysioNet [27,31]. Another important attribute of noise being investigated is the noise level, which is achieved by controlling the SNRs when adding the three noise types to the ECG waveforms using the WFDB software. A wide range of SNR levels (i.e., −6, 0, 6, 12, 18, and 24) is included in the study to evaluate the algorithm’s performance against six levels of noise in the signal. A two-way ANOVA test is performed to analyze the effect of the noise level and the noise type on the algorithm performance. Simple main effects analyses are performed if there exists a significant interaction between the two noise factors. Tukey’s HSD tests are also performed for any of the two factors that are significantly associated with the algorithm performance. 

#### 2.4.4. Experiment #4 Sampling Rate Test

The signal sampling rate may vary from device to device or within the same device with different settings, due to technical considerations such as storage capacity and energy consumption. Therefore, it is important to test whether the signal sampling rate is a significant factor impacting the algorithm performance. In the fourth experiment, two more sets of samples are generated by downsampling the sample set with the sampling rate of 250 Hz to 200 Hz and 150 Hz, respectively. A one-way ANOVA test is then performed to compare the algorithm performance of the three sample sets and determine whether the sampling rate is significantly associated with the algorithm performance.

#### 2.4.5. Experiment #5 Comparison to DTW-Based Approach

In this experiment, the second component of the framework (i.e., calculation of cross-correlation) is swapped with a DTW-based algorithm, which estimates RR intervals of each ECG signal as in [23] and uses DTW on the time series of RR intervals to determine the time alignment between the two ECG signals. Other components of the framework are left unchanged so that a direct performance comparison between the proposed approach and the DTW-based approach can be obtained. A two-sample Student’s *t*-test is performed to statistically compare the algorithm performance from the two approaches.

## 3. Results

**Figure 1:**
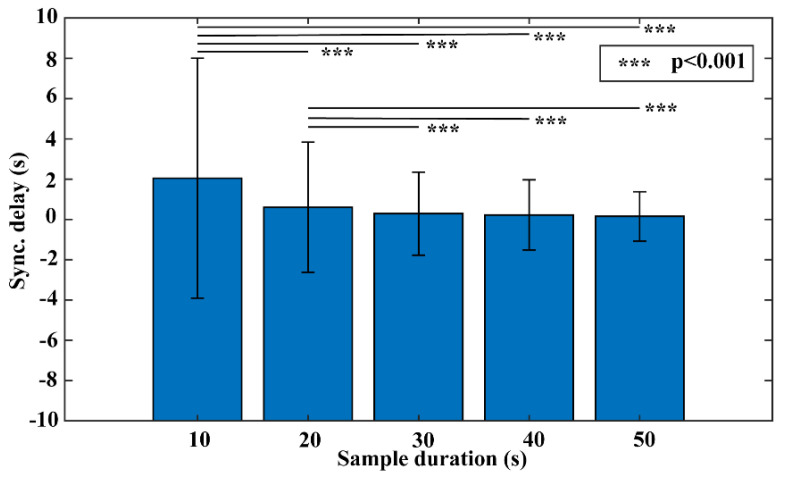
The association between sample durations and time-alignment algorithm performance.

Figure 1 presents the algorithm performance in sync delay with different options of sample durations for the Lead I samples. It shows that the performance consistently improves with increasing sample durations. It also presents a reduced variation in performance as the sample duration increases. The one-way ANOVA test shows a significant association between sample duration and algorithm performance (F = 443.01, *p* < 0.001). The worst sync delay is 2.05 s with the sample duration at 10 s, which is greatly improved to 0.15 s when a sample duration of 50 s is used. The post-hoc Tukey’s HSD tests show that there are significant differences between performance with sample durations below (including) 20 s and above (including) 30 s (*p* < 0.001). It shows that there is no significant difference in performance with the sample duration exceeding (including) 30 s (*p* > 0.05). The sample duration at 30 s is therefore selected for subsequent experiments, as it strikes a balance between algorithm performance (mean sync delay mean: 0.29 s) and efficiency that requires a shorter collection time than the other two good-performing sample duration options (i.e., 40 s and 50 s).

**Figure 2:**
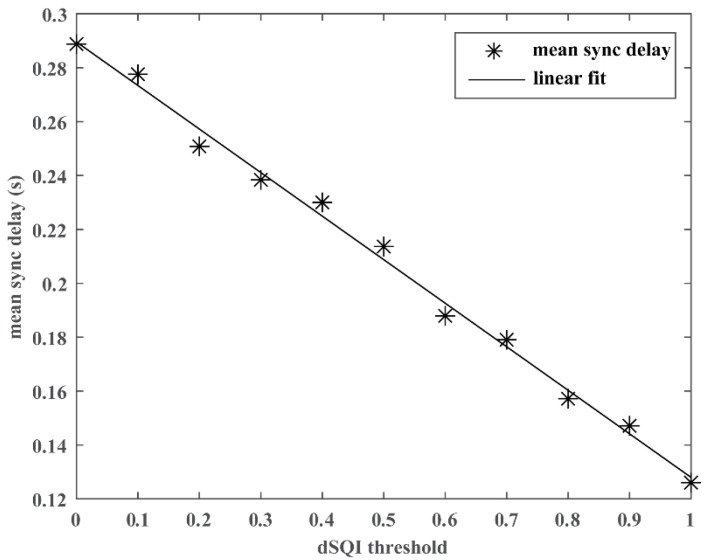
Correlation between dSQI thresholds and algorithm performance.

Table 1 summarizes the algorithm performance in sync delay when integrating various SQI thresholds to remove samples with relatively poor signal quality. It shows that the performance consistently improves as a more stringent SQI threshold is implemented. The mean sync delay drops from 0.29 s with an SQI threshold of 0 (i.e., no SQI filtering) to 0.13 s with an SQI threshold of 1 (i.e., retaining perfect quality samples). As shown in Figure 2, the Pearson correlation analysis shows that the performance strongly correlates with the SQI threshold with a statistical significance (correlation coefficient r = −0.997, *p* < 0.001). It is worth noting that even with the strictest SQI threshold at 1, there are still 43% of samples fulfilling the criteria. The following three experiments are carried out with a fixed SQI threshold set at 1. 

**Figure 3:**
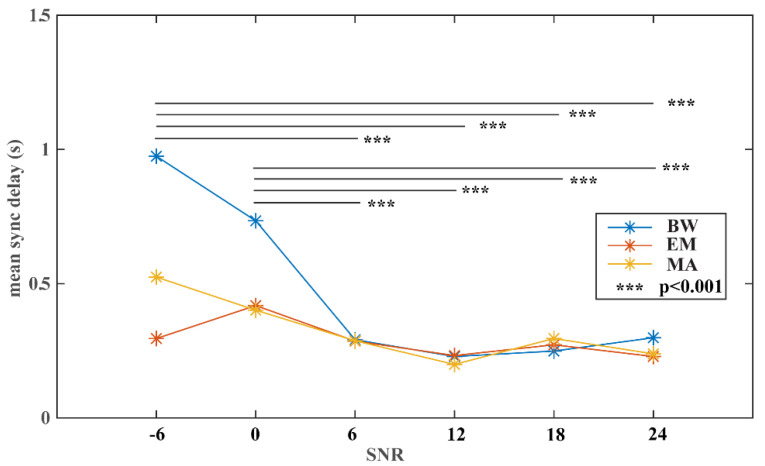
Time-synchronization algorithm performance with different noise types and noise levels.

Figure 3 depicts the changing profiles of performance along with varying noise levels in the signal for the three common noise types (i.e., BW, EM, and MA). It presents a generally decreasing trend in mean sync delay with increasing SNRs of the signal across all noise types, indicating that the algorithm suffers from performance loss when noise is present in the signal. Since two key factors exist with respect to the signal noise, i.e., the noise type and the noise level, a two-way ANOVA test can reveal whether there is a significant interaction between the two independent variables. The test result shows that there is a significant interaction effect between the noise type and the noise level (F = 3.51, *p* < 0.001). The follow-up simple main effects tests show that there exists a significant association between the noise type and the algorithm performance (F = 4.49, *p* < 0.05), and there is a much stronger association between the noise level and the algorithm performance (F = 13.99, *p* < 0.001). As also shown in Figure 3, there exists a pivotal signal quality level, with SNRs lower than 6 leading to significantly worse sync delays than higher ones (Tukey’s HSD test, *p* < 0.001) and no significant difference in the performance when SNRs are higher (including) than 6. When comparing different noise types, pairwise comparisons with Tukey’s HSD test show a statistical difference in performance only between the BW noise and the EM noise (*p* < 0.05). 

**Figure 4:**
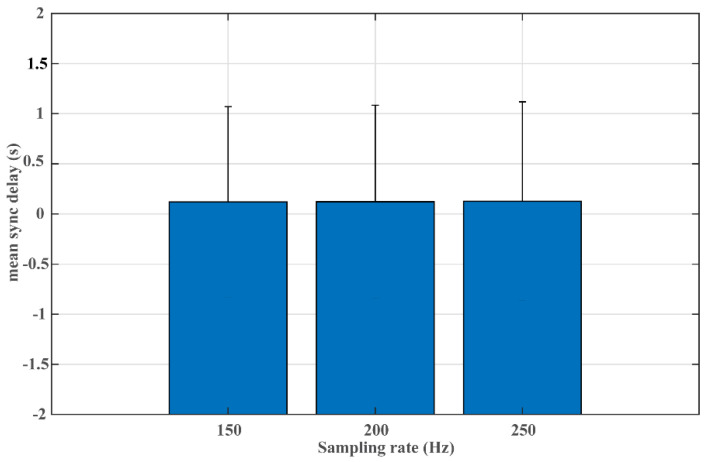
Association of sampling rate with the algorithm performance.

The impact of the sampling rate on the algorithm performance is presented in Figure 4. When varying the sampling rate from 150 Hz to 250 Hz, a similar performance level is presented with mean sync delays at 0.12 s, 0.12 s, and 0.13 s for sample sets with sample rates at 150 Hz, 200 Hz, and 250 Hz, respectively. This is further confirmed with the one-way ANOVA test, which shows no significant association between the sampling rate and the model performance (F = 0.03, *p* = 0.966). 

When adopting the same sample duration of 30 s and quality index threshold of 1, the DTW-based approach achieves a mean sync delay at 1.44 s (SD: 4.59 s), which is significantly higher than the performance of the proposed algorithm using temporal cross-correlation (mean: 0.13 s; SD: 0.99 s; *p* < 0.001). 

## 4. Discussion

The present study proposed an effective time-matching approach based on the temporal cross-correlation of a common signal type available across different devices. Achieving the precise time alignment of signals has great practical implications, as it offers a solution to link together multimodal data across different devices. It is also the cornerstone to evaluate the signal fidelity of novel wearables against established devices as the field of digital health is steadily moving toward portable and wearable setups. A multi-lead public ECG database (i.e., INCART) with long-term recordings and a wide spectrum of ECG abnormities was adopted to simulate the time-matching scenario of single-lead ECGs from an ECG patch and from an ECG watch, both of which are commonly used in today’s wearable technology landscape. The dataset was selected because simultaneously recorded multi-lead ECG can readily provide ground truth alignment to validate the algorithm performance. In addition, the data were collected from patients with heterogeneous cardiac conditions, making it a great choice to assess the algorithm’s efficacy in close to real-world scenarios. The study took into consideration key technical and environmental factors, including sample durations, SQI integration, resilience to noise, and varying sampling rates, to comprehensively evaluate the performance of the proposed time-matching approach and make corresponding recommendations. 

When comparing performance from five different sample durations, the performance generally improves when a longer duration of data is available for the time-alignment algorithm. Such an observation is expected, as the centerpiece of the algorithm calculates the temporal cross-correlation between two signals, which essentially evaluates the agreement of temporal signal patterns between the two. When longer signals are provided, the algorithm has more patterns to work with, resulting in improved performance (as demonstrated by the shorter sync delay in Figure 1) and stability (as demonstrated by the smaller standard deviation in Figure 1). However, the proposed time-matching approach aims to strike a balance between performance and practicality. The performance gain from longer sample durations comes with the burden of a long sample collection time for the algorithm to operate. In the ECG patch-watch scenario, it means that the users would need to touch an ECG electrode for a longer period to collect the spot-check ECG signal from the ECG watch, imposing considerable inconvenience to users. Longer sample duration also translates to higher demand on computing capacity and energy consumption, both of which are parsimonious in wearables, as the development is trending toward a small form factor for lightweight and portability. Results in Figure 1 demonstrate that a sample duration of 30 s significantly improves the algorithm performance over 10 s (*p* < 0.001) by reducing the mean sync delay from 2.05 s to just under 0.3 s. Further increasing the sample duration to 40 s and 50 s does not offer significant performance improvement over the 30 s (*p* > 0.05). The observation suggests 30 s as the recommended sample duration for the proposed time-matching algorithm. Interestingly, it is the same duration adopted by many popular ECG watches currently available on the market, such as Apple Watch (Apple Inc., Cupertino, CA, USA), Samsung Galaxy Watch (Samsung Electronics, Seoul, Korea) and Fitbit Sense (Fitbit, Inc., San Francisco, CA, USA). 

Digital health innovations bring about great benefits and opportunities, such as enabling simple and flexible data collection to accelerate telehealth [32], mapping out a more complete picture of patient physiological conditions from patient-generated health data [33,34], and promoting precision medicine [35]. Meanwhile, some existing challenges in acquiring physiological data can be exacerbated, among which signal quality is one of the most prominent issues. Taking wrist-based PPG as an example, one study evaluated wrist-based PPG acquired over a 24 h period and found only 45% of the signals were of high quality [14]. The issue is largely contributed by the motion artifact that is pervasive in signals collected with wearables due to their ambulatory nature [36,37]. The two-step approach implemented in the present study demonstrates great efficacy in curbing the signal quality issue. A series of preprocessing steps are first applied to mitigate common noises in ECG and prep the signal for the subsequent time-matching algorithm. Next, an overall quality index, dSQI, is derived to gauge the signal quality of the entire sample so that different quality levels can be used as thresholds to filter out poor quality samples from time-matching analysis. As shown in Figure 2, there is a significant correlation (r = −0.997, *p* < 0.001) between time-matching precision and dSQI thresholds, with consistent improvement in mean sync delays along with the tightening requirement of signal quality. With the integration of dSQI filtering, the mean sync delay can be further reduced from 0.29 s to 0.13 s using the threshold of 1, while still retaining 43% of all samples (see Table 1). During real-world implementation, this step can be readily translated into a rule to determine whether acquired signals surpass the minimal quality requirement for the algorithm and whether a repeated measure is needed to obtain a valid sync delay. It is worth noting that the experiments were simulated based on long-term recordings, which were acquired with medical devices in clinical settings. The operating dSQI threshold should be recalibrated when there is a change in acquisition devices, signal types, or acquisition environment to achieve optimal outcomes from the time-matching algorithm. 

The noise stress test is a great way to learn the algorithm performance on signals collected in dynamic and complex free-living conditions. Therefore, we put the time-alignment algorithm under a comprehensive noise stress test by varying across three common noise types in ECG (i.e., BW, EM, and MA) and a broad range of noise levels. A significant interaction effect was discovered between the two factors, indicating different noise types impact the algorithm performance differently when different levels of noise are present in the signal. After disentangling the two factors, it shows three common noise types affect differently on the algorithm performance, with most of the differences contributed by the discrepancy between EM and BW. More importantly, it reveals that a pivotal noise level can be obtained, with SNR at 6 dB as the lower bound for the algorithm to perform stably and well.

With the rapidly growing catalog of digital health devices, many are operated with different sampling rates depending on the use scenarios and with consideration of battery conservation. Taking ECG-based heart rate variability (HRV) estimation as an example, although it is recommended to record ECG at a sampling rate of at least 250 Hz without interpolation [38], numerous later studies reported that similar HRV can be obtained with a lower sampling rate. Ellis et al. found that ECG collected at 125 Hz is good enough for HRV estimation without compromising accuracy [39]. Ziemsen et al. found no statistically significant differences in HRVs derived from ECG recorded at 100 Hz, 200 Hz, and 500 Hz [40]. In one recent study, Kwon et al. made a suggestion to adopt 250 Hz as the sampling rate for HRV analysis, while outlining that 100 Hz is also acceptable if no spectral analysis is required [41]. Therefore, it is necessary to evaluate the time-matching algorithm with ECG signals of different sampling rates. The high concordance in performance achieved by sampling rates of 150 Hz, 200 Hz, and 250 Hz in the present study (see Figure 4) demonstrates the high adaptability of the proposed time-matching approach to the varying sampling rates in digital health devices. It is worth noting that the study leverages a multi-lead ECG dataset, as it provides ground truth for the time alignment. However, ECG waveforms from different leads share the same original sampling rate, making it infeasible to test the algorithm performance on signals of different original sampling rates in this study. Although the proposed framework can resample signals to a common sampling rate and the results from Experiment #4 demonstrate stable algorithm performance across varying sampling rates, it requires additional validation on the distinct-sampling-rate scenario when data become available. 

Unlike other studies that directly tackle the time alignment of two different types of physiological signals [22,23], the present study aims to solve a specific time-matching problem when common signal types are available across devices. Compared to other studies, the core time-matching algorithm in the proposed approach only involves light computation of temporal cross-correlation between two signals to determine the optimal time alignment, which is energy efficient and in favor of edge computing. Using the common data type as the bridge to synchronize multimodal data also avoids the singularity issue plaguing the time-matching tasks, as signals of the same type collected from different devices can be readily resampled to the same frequency to achieve a one-to-one matching scheme. In addition, the proposed algorithm operates on amplitudes of the signal without the need for identifying signal landmarks, extra events, or any encoding process of the signals, which themselves are prone to introducing additional errors into the time-matching performance. This also makes the algorithm agnostic to signal types so that it can be readily applied to other common physiological signals acquired in free-living settings, such as PPG, electrodermal activity signal, etc. Results from Experiment #5 reveal a significantly longer delay obtained by the more complex DTW-based approach, which provides additional support for the use of a simple cross-correlation algorithm in tackling this specific time-alignment task with two signals of the same signal type. 

The proposed algorithm is comprehensively evaluated against common factors involved in the time-matching process, including sample duration, integration of signal quality index, tolerance to noise, and varying sampling rates. We determine that a sample duration of 30 s strikes a good balance between algorithm performance and user convenience, and the algorithm can offer certain tolerance to varying sampling rates by providing comparable performance across different sampling rates tested. Our results also demonstrate the improved performance with the integration of an SQI threshold as the minimal requirement of signal quality to trigger the time-matching algorithm. The optimal threshold needs to be recalibrated for different signal types, and one should take into consideration the extra burden imposed by the thresholding step. When comparing time-matching performance with different noise types and noise levels presented in the signal, we find noise level to be the most critical factor impacting the model performance. In our noise stress test, the algorithm can offer a stable and reasonable performance after a pivot noise level (i.e., SNR at 6 dB), which can serve as the minimal operatable point for the algorithm to provide valid outputs. 

One limitation of the algorithm lies in its requirement of a common signal type across devices. Nonetheless, the study is tailored to complement existing time-matching algorithms that are designed to align different signal types, by leveraging the common signal modality when available across devices to obtain cross-device time synchronization. The demonstrated performance from the time-matching algorithm has great implications in validating emerging wearable or wireless sensor data against those from established devices, as well as fusing multimodal signals from multiple devices for downstream clinical applications. There are several future directions that are worth further pursuing. First, the present study uses signals from 2 of 12 ECG leads in the public ECG database to simulate the ECG watch–patch setup. Future effort in validating the algorithm to data collected in free-living settings is warranted. Second, the algorithm is designed to be agnostic to the common signal type, while the scope of the study has been so far limited to using ECG as the common signal type. Additional validation of the algorithm performance is still needed to establish its efficacy on other signal types. Third, the INCART database offers ECG recordings in 30 min durations, whereas there exist other databases, such as the long-term ST database [42], that offer all-day or even multi-day long-term ECG monitoring and can be leveraged to validate the long-term stability of the algorithm. Fourth, a great body of knowledge exists in the research field of wireless sensor networks that deals with the time synchronization of data from an array of individual sensors [43,44,45]. It is worthy of further investigation whether some of these methods can be adopted for synchronizing physiological signals and how the performance stacks up to the proposed algorithm. Lastly, one practical factor worth further exploring is how often to initiate the time-matching procedure, which is complex due to varying temporal delays across different devices but carries great implications in estimating the end-user workload and streamlining the time-matching process in real-world settings.

## Figures and Tables

**Table 1:** Time-synchronization algorithm performance with varying dSQI thresholding.

dSQI Threshold	Sync Delay (Mean/SD, Seconds)	Sample Retaining Ratio (%)
0.00	0.29/2.06	1.00
0.10	0.28/2.00	0.94
0.20	0.25/1.82	0.92
0.30	0.24/1.75	0.89
0.40	0.23/1.70	0.86
0.50	0.21/1.61	0.82
0.60	0.19/1.40	0.78
0.70	0.18/1.34	0.73
0.80	0.16/1.13	0.68
0.90	0.15/1.08	0.61
1.00	0.13/0.99	0.43

## Data Availability

The data presented in this study are openly available in PhysioNet at https://doi.org/10.13026/C2V88N, reference number [27].

## Supplementary Materials

The following supporting information can be downloaded at: https://www.mdpi.com/article/10.3390/jimaging8050120/s1, Table S1: Notation and Abbreviation Index Table.
