# Supplementary material for: Time Synchronization of Multimodal Physiological Signals through Alignment of Common Signal Types and Its Technical Considerations in Digital Health
Source: J Imaging. 2022 Apr 21;8(5):120. doi: 10.3390/jimaging8050120 (PMC9145353; doi:10.3390/jimaging8050120)
Supplement: Supplementary file 1 [file jimaging-08-00120-s001.zip › jimaging-1640371-supplementary.pdf]

Table S1. Notation and Abbreviation Index Table.

Notation	Description
S_I	ECG signal from Lead I
S_{V2}	ECG signal from Lead V2
t_i	The time lag t introduced to S_{V2} at ith step
$\tilde{S}_{V2_i}$	The time shifted ECG signal from Lead V2 by the time lag t_i
r_i	The correlation between Lead I and time-shifted Lead V2 signals by the time lag t_i
t_{max}	The time lag with maximal correlation between two signals
τ	Optimal time alignment determined by the proposed algorithm
**Abbreviation**	**Description**
BW	Baseline wandering
dSQI	Dynamic signal quality index
DTW	Dynamic time warping
EM	Electrode motion artifact
INCART	The St. Petersburg Institute of Cardiological Technics
MA	Muscle artifact
SQI	Signal quality index
